# Congenital fibrosis of the extraocular muscles

**DOI:** 10.4103/0974-620X.71912

**Published:** 2010

**Authors:** Abdullah Al-Mujaini

**Affiliations:** Department of Ophthalmology, College of Medicine and Health Science, Sultan Qaboos University, Muscat, Sultanate of Oman

Sir,

I read with great interest the article tilted, “Congenital fibrosis of the extraocular muscles (CFEOM)” by Cooymans *et al*.[1] Although the authors have briefly mentioned the genetic basis underlying this condition, this section merits elaboration, as it sheds light on the pathogenesis of this complex condition.

Current scientific thought is that these clinical entities have an underlying genetic cause. Regardless of clinical variations, they are being grouped first by genetic etiology and then by clinical differences. A major dilemma lies in its pathogenesis, but recent genetic studies support the hypothesis of aberrant development of motor nuclei in midbrain and pons. Despite the fact that diagnosis can be defined by clinical characteristics as well as genetic study, this is not an easy task to apply all the time. Dynamic MRI imaging is a valuable adjunct in the clinical evaluation of CFEOM.[2] Orbital imaging of this condition demonstrates extraocular muscles and motor nerve hypoplasia.

To date, four CFEOM genotypes have been described. They include CFEOM1, CFEOM2, CFEOM3, and Tukel syndrome. The locus of CFEOM 1 is located at the centromere of chromosome 12. The transmission is usually autosomal dominant (AD) with complete penetrance. It is associated with the absence of superior division of cranial nerve III and corresponding midbrain motor neurons as well as profound atrophy of levator and superior rectus muscles.[3] CFEOM1 can be autosomal recessively (AR) transmitted suggesting this second recessive locus may be allelic to the AD CFEOM1 locus.[4] The mutation for this type is a heterozygous missense mutation located in a kinesin motor protein coded by KIF21A. There are six different mutations in 44 of 45 probands. The primary mutational hot spots in the stalk domain suggests an important new role for *KIF21A* in the formation of oculomotor axis.[5] CFEOM2 is an AR disorder mapping to chromosome 13. Developmental insult to both the superior and inferior divisions of the oculomotor and trochlear nerves is hypothesized, although no pathological specimens have been evaluated. Three mutations in *ARIX (PHOX2A)* in four CFEOM2 pedigrees have been identified. *ARIX* required for CN III and CN IV development in mouse and zebrafish, confirms the hypothesis that CFEOM2 results from abnormal CN III and IV development.[6] CFEOM3 is an AD disorder mapping to CFEOM3 locus on chromosome 16q24. It has a wider range of clinical characteristics related to variable involvement of the third cranial nerve.[7] Recently, *TUBB3* has been described as the gene responsible for CFEOM3.[8] Tukel syndrome is a restrictive ophthalmoplegia with blepharoptosis and postaxial oligodactyly/oligosyndactyly of the hands. A genomewide scan established linkage to a locus on chromosome 21qter.[9]
